# A Novel Dual Bruton's Tyrosine Kinase/Janus Kinase 3 Inhibitor Wj1113 and its Therapeutic Effects on Rheumatoid Arthritis

**DOI:** 10.1002/mco2.70207

**Published:** 2025-07-07

**Authors:** Chunyu Zhang, Fangfang Lai, Hang Gong, Shuying Li, Nan Xiang, Liuyi Que, Nina Xue, Mengyao Hao, Enjia Zhou, Xiaojian Wang, Taigang Liang, Jing Jin

**Affiliations:** ^1^ State Key Laboratory of Bioactive Substances and Functions of Natural Medicines Institute of Materia Medica Chinese Academy of Medical Sciences and Peking Union Medical College Beijing China; ^2^ School of Pharmacy Medicinal Basic Research Innovation Center of Chronic Kidney Disease Ministry of Education Shanxi Medical University Taiyuan China; ^3^ Collaborative Innovation Center of Advanced Drug Delivery System and Biotech Drugs in Universities of Shandong Key Laboratory of Molecular Pharmacology and Drug Evaluation (Yantai University) Ministry of Education Yantai University Yantai China; ^4^ Department of Oncology The Second Hospital of Shanxi Medical University Taiyuan China

**Keywords:** BTK, CIA, dual inhibitor, JAK3, RA

## Abstract

Rheumatoid arthritis (RA) is a chronic autoimmune disease characterized by joint inflammation and tissue damage, driven by dysregulated cytokine signaling and immune cell hyperactivation. Bruton's tyrosine kinase (BTK) mediates pathogenic B‐cell activation and autoantibody production, while Janus kinase 3 (JAK3) orchestrates cytokine‐driven inflammation through signal transducer and activator of transcription 5 (STAT5) phosphorylation, exacerbating macrophage and monocyte activation. Here, we report Wj1113, a novel dual inhibitor that potently blocks BTK (IC_50_ = 0.7 nM) and JAK3 (IC_50_ = 26.2 nM). Wj1113 inhibits B‐cell activation via BTK blockade and suppresses JAK3‐dependent STAT5 phosphorylation, reducing proinflammatory cytokine secretion and monocyte chemotaxis. In vitro, it suppresses macrophage activation and modulates inflammatory mediator expression. In the collagen‐induced arthritis mouse model, Wj1113 treatment dose‐dependently reduces joint inflammation, macrophage infiltration, and levels of TNF‐α (tumor necrosis factor‐α), IL(interleukin)‐6, anti‐cyclic citrullinated peptide antibody (ACPA) and rheumatoid factor (RF), while elevating anti‐inflammatory IL‐10. Histopathological and micro‐CT analyses confirm attenuation of cartilage/bone erosion and synovial hyperplasia. Mechanistically, Wj1113 inhibits BTK/JAK3 signaling in vivo and alleviates arthritis in joints. Collectively, these findings establish Wj1113 as a promising dual‐target therapeutic candidate for RA, addressing both B‐cell and cytokine‐driven pathogenic pathways.

## Graphical abstract

Wj1113 simultaneously inhibits the JAK3 and BTK pathways, impacting the activation of macrophages and B cells, as well as influencing monocyte chemotaxis. Consequently, this results in a decrease in proinflammatory factors such as MCP‐1(monocyte chemoattractant protein‐1), G‐CSF(granulocyte colony‐stimulating factor), IL‐6, TNF‐α, and RANTS(regulated on activation, normal T‐cell expressed and secreted) within the affected joints. Additionally, Wj1113 increases the anti‐inflammatory factor IL‐10. Therefore, Wj1113 has the potential to alleviate the pathology of rheumatoid arthritis.

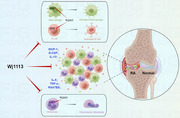

## 1 Introduction

Arthritis, a chronic inflammatory disorder, is characterized by joint inflammation, pain, and the progressive destruction of cartilage and bone. This condition affects millions of individuals globally, imposing a substantial burden on patients’ quality of life and healthcare systems [1, 2]. Current treatment strategies for arthritis mainly encompass the use of small molecule disease‐modifying antirheumatic drugs (DMARDs), corticosteroids, and biologics. Small molecule DMARDs like methotrexate and corticosteroids are frequently employed to manage the disease. Nevertheless, their long‐term administration is accompanied by various adverse side effects. For instance, immunosuppression and organ toxicity are common concerns associated with their extended use [3, 4]. Biologics, such as anti‐TNF‐α and anti‐CD20 （cluster of differentiation 20） antibodies, have demonstrated encouraging efficacy in alleviating inflammation and impeding disease progression [5]. However, the clinical response rates remain less than optimal, and a significant number of patients fail to achieve complete remission. Thus, there is an urgent imperative to uncover safer and more effective therapeutic approaches for arthritis treatment [6].

The pathogenesis of arthritis is a multifaceted process involving intricate immune responses. Cytokine signaling dysregulation, abnormal immune cell activation, and autoantibody production all contribute to the development and progression of this disease [7]. Macrophages, for instance, play a central role. They migrate into joint tissues, become hyperactivated, and drive osteoclast differentiation, which in turn enhances bone resorption [3, 8]. This excessive osteoclastic activity contributes to the progressive joint destruction seen in arthritis. Monocytes also significantly contribute to arthritis progression. Their chemotaxis toward inflamed joints is a key event. Once recruited, monocytes differentiate into macrophages and osteoclasts, secreting high levels of proinflammatory cytokines and exacerbating tissue damage [9]. Additionally, B cells are key players in the pathogenesis of arthritis, primarily by producing autoantibodies that form immune complexes, leading to complement activation and inflammation, further exacerbating joint damage [10]. Given their pivotal roles, targeting both macrophages and B cells has become a major focus in arthritis research.

In recent years, several promising therapeutic targets have emerged in arthritis research. Among these, Bruton's tyrosine kinase (BTK) and Janus kinase 3 (JAK3) have garnered significant attention [11, 12]. BTK is essential for B cell receptor signaling, and JAK3 mediates cytokine receptor signaling, especially for the common γc cytokines [13, 14]. However, single‐target inhibitors face inherent limitations. They often fail to comprehensively address the complex immune dysregulation in arthritis and are prone to resistance development [15]. To address these challenges, there has been growing interest in dual BTK/JAK3 inhibitors. To overcome these challenges, dual BTK/JAK3 inhibitors have emerged as a promising approach. These compounds have the potential to simultaneously modulate macrophage and B cell activation, monocyte chemotaxis, and cytokine signaling pathways, offering a more comprehensive treatment strategy [16, 17].

In our pursuit of novel therapeutic agents, we identified compound Wj1113 through an integrated drug design approach. Initially discovered as a potent BTK inhibitor capable of significantly suppressing the proliferation of B‐cell lymphoma [18], comprehensive kinase profiling further revealed its robust inhibitory activity against JAK3. This dual inhibitory property makes Wj1113 a promising candidate for treating rheumatoRA).

Against this backdrop, this study aims to comprehensively explore the therapeutic potential of Wj1113 in arthritis treatment. Specifically, we seek to understand how Wj1113 impacts cytokine signaling, immune cell activation, and joint inflammation, all of which are pivotal in the etiology and progression of RA. Additionally, we will evaluate its efficacy in the commonly used collagen‐induced arthritis (CIA) model. Our research is expected to provide valuable insights into its therapeutic effectiveness, mechanisms of action, and contribute to the development of more targeted and efficient arthritis therapies.

## 2 Results

### 2.1 The Specific Inhibition of Wj1113 Against BTK and JAK3 Kinases and its Underlying Molecular Mechanisms

**FIGURE 1:**
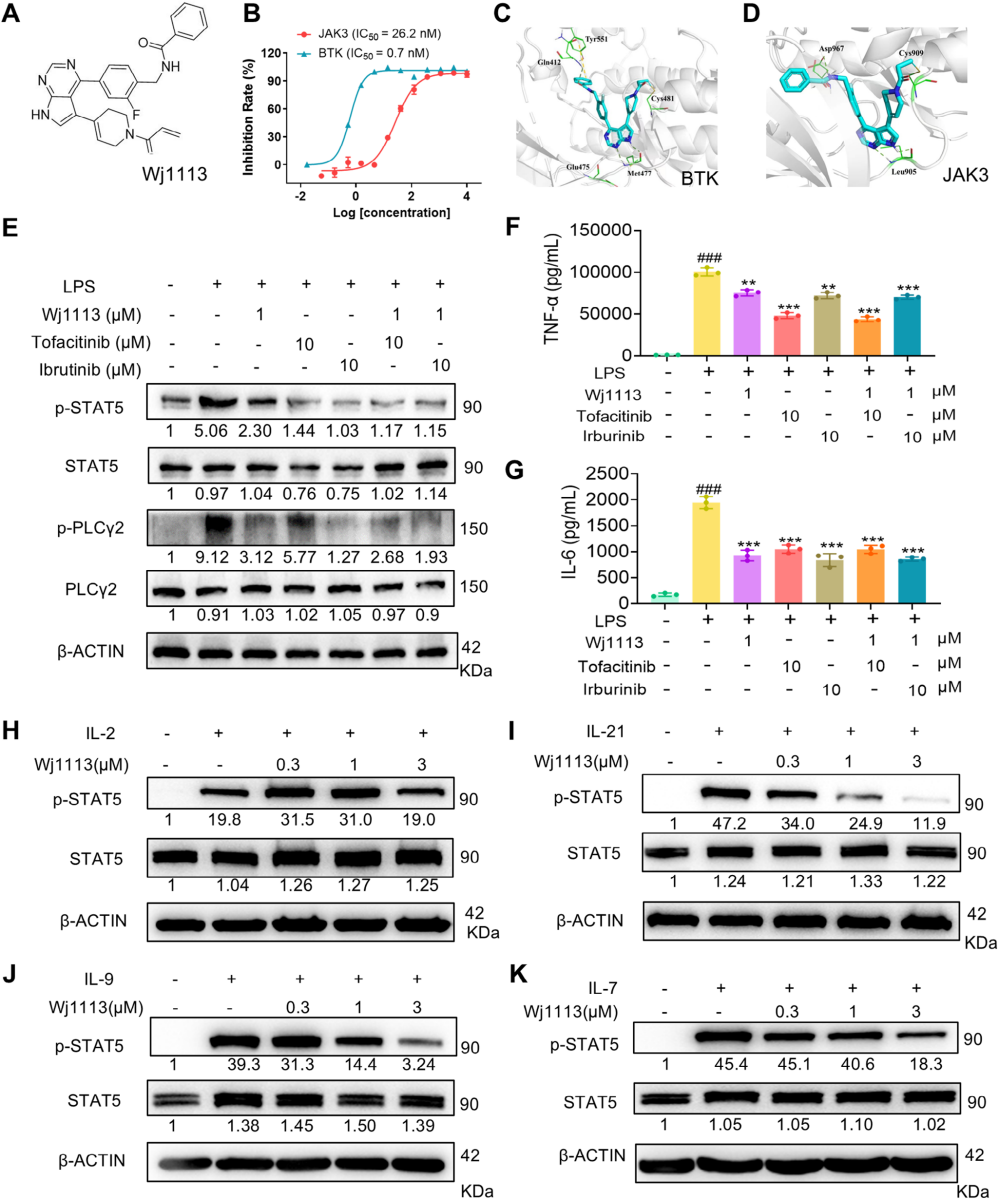
Effects of Wj1113 on BTK/JAK3. (A) Chemical structure of Wj1113. (B) Inhibitory activities of Wj1113 on JAK3 and BTK kinases. (C and D) Covalent docking interactions of Wj1113 with the BTK and 1macrophages pretreated with tofacitinib and ibrutinib for 1 h, followed by incubation with Wj1113 for an additional 24 h. (F and G) Effects of Wj1113 on TNF‐α and IL‐6 levels in the cell supernatants obtained from the experiment E. (H–K) Influence of Wj1113 on the JAK3 downstream signaling pathway. Different concentrations of Wj1113 were incubated with mouse splenic lymphocytes for 1 h, and then 20 ng/mL of cytokines were added for 15 min of stimulation, followed by Western blot detection of p‐STAT5 expression. Data are from three independent experiments. All data are presented as means ± standard deviation (SD). Statistical significance is indicated as follows: ###*p* < 0.001 versus the control group; **p < 0.01, and ***p < 0.001 versus the cytokine treated group.

Previously, it was demonstrated that Wj1113 effectively inhibited BTK kinase activity with an IC_50_ of 0.7 nM [18]. Through comprehensive kinase profiling analysis, it was further found that Wj1113 exhibited a potent inhibitory effect on JAK3 with an IC_50_ of 26.2 nM (Figure 1A,B).

To elucidate the binding mode and strength of Wj1113 with BTK and JAK3, molecular docking was conducted. The results revealed that Wj1113 formed a covalent bond with Cys481 in BTK. Its pyrrolopyrimidine core interacted with Met477 in the hinge region via hydrogen bonds, and the phenol group occupied the selective H3 pocket near Tyr551 (Figure 1C). Similarly, Wj1113 formed a covalent bond with Cys909 in JAK3, with hydrogen bonds between the pyrrolopyrimidine core and Leu905, and an amide group interacting with Asp967 (Figure 1D). These findings strongly suggested a robust covalent binding of Wj1113 to both BTK and JAK3.

To validate the target specificity of Wj1113, specific inhibitors targeting JAK3 and BTK were utilized to suppress the activity of the corresponding kinases. As shown in Figure 1E, pretreatment with high doses of JAK3 or BTK inhibitors in the RAW264.7 macrophage completely inhibited the phosphorylation of their respective downstream signaling proteins, phospholipase Cγ2 (PLCγ2) (for BTK) and STAT5 (for JAK3). After this suppression, treatment with Wj1113 did not further decrease the phosphorylation levels of either protein, confirming that Wj1113 acts specifically by inhibiting BTK and JAK3 activity. Moreover, when analyzing the secretion of inflammatory cytokines in the cell supernatants, a similar trend was observed. Pretreatment with JAK3 or BTK inhibitors significantly reduced macrophage secretion of TNF‐α and IL‐6. Subsequent addition of Wj1113 did not result in further reduction of these cytokines (Figure 1F,G).

The JAK3 subtype specifically responds to IL‐2, IL‐4, IL‐7, IL‐9, IL‐15, and IL‐21 cytokine receptors [19, 20]. To further confirm the inhibitory effect of Wj1113 on JAK3 protein, we added cytokines IL‐2, IL‐7, IL‐9, and IL‐21 to activate the JAK3‐STAT5 signaling pathway in mouse splenic lymphocytes and used western blot analysis to detect the effects of the compound on downstream p‐STAT5. As shown in Figure 1H–K, the expression of p‐STAT5 increased significantly upon the addition of these cytokines. Wj1113 effectively inhibited the phosphorylation of STAT5 in a dose‐dependent manner starting from 0.3 µM. These results further indicated that Wj1113 could effectively block JAK3–STAT5 signaling pathway activated by cytokines [19, 20].

### 2.2 Wj1113‐Mediated Inhibition of Monocyte Chemotaxis

**FIGURE 2:**
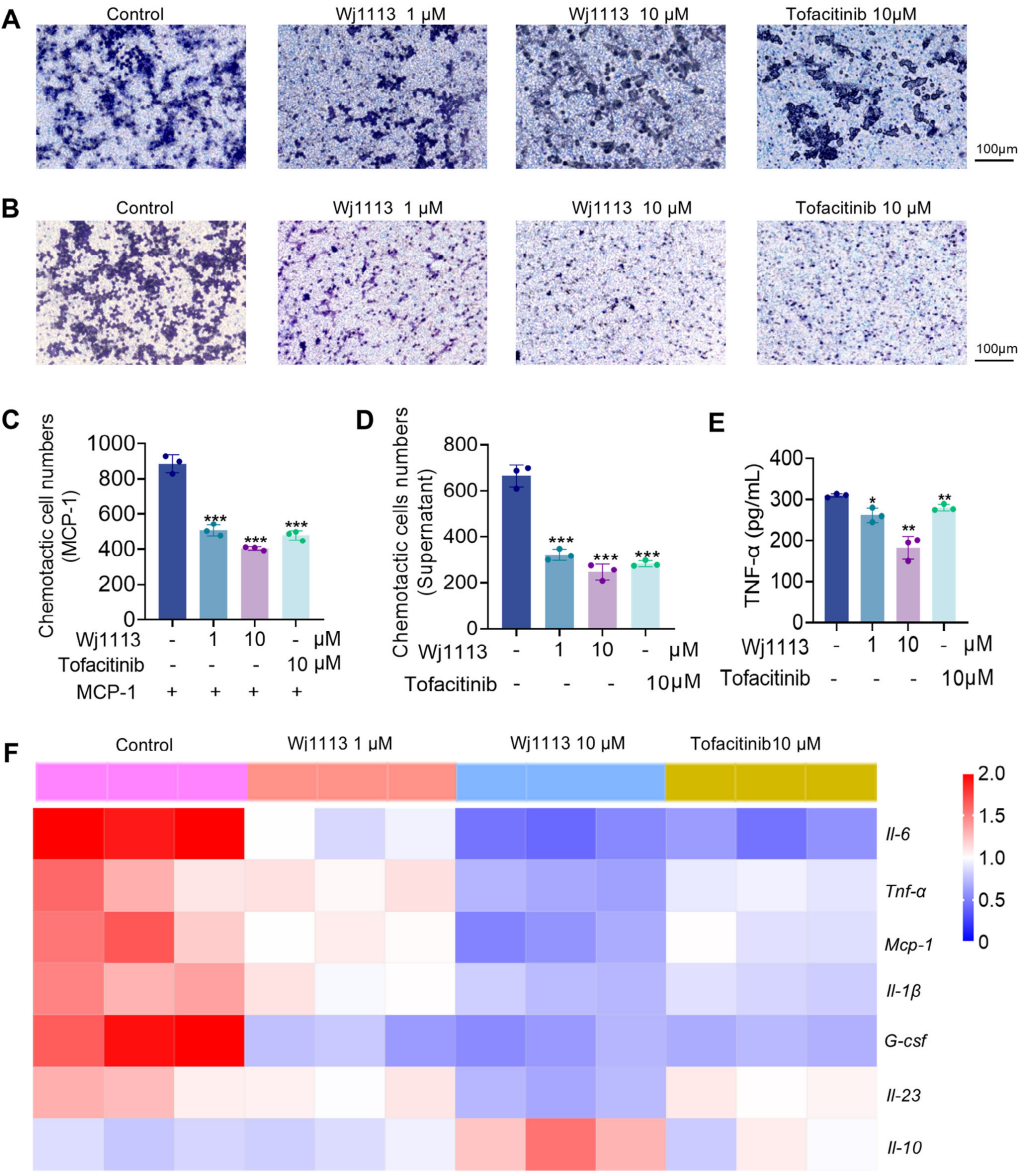
Effects of Wj1113 on THP‐1 chemotaxis. (A and C) Inhibitory effects of Wj1113 on MCP‐1‐induced THP‐1 chemotaxis. THP‐1 cells were pretreated with different concentrations of Wj1113 for 48 h, then seeded into transwell chambers, and 250 ng/mL MCP‐1 in the lower chamber was used to stimulate for 3.5 h. (B and D) Chemotactic effects of supernatants from Wj1113‐treated cells on THP‐1. Untreated THP‐1 cells were seeded into transwell chambers, with the lower chamber filled with supernatants from the experiment in A for 24 h. For both (A and B) scenarios, after incubation, migrated cells on the lower membrane were stained with crystal violet, photographed, and counted. (E) TNF‐α levels in the lower chamber supernatants from A were measured by ELISA. (F) Heatmap presenting the statistical effects of Wj1113 on the mRNA expression levels of different cytokines. Migrated cells in the lower chamber from the experiment in A were collected for RT‐qPCR experiments, repeated three times, and drew a heatmap using GraphPad Prism 8.0. Data from three independent experiments are presented as means ± SD. Statistical significance is indicated as follows: *p < 0.05, **p < 0.01, and ***p < 0.001 versus the control group.

Monocytes play a crucial role in arthritis pathogenesis, with their activation and chemotaxis being tightly linked to JAK3 signaling. Aberrant JAK3 activation can lead to excessive monocyte recruitment and activation, thereby aggravating joint inflammation. In this study, we initially explored the capacity of Wj1113 to counteract MCP‐1‐induced monocyte chemotaxis. Utilizing THP‐1 cells, we observed that treatment with Wj1113 at a 1 µM concentration notably reduced monocyte migration (Figure 2A,C). Subsequently, we investigated the impact of Wj1113 on monocyte behavior when induced by normal cell culture supernatant without MCP‐1(Figure 2B,D). The results revealed that Wj1113 exerted a more pronounced inhibitory effect on monocyte migration. Remarkably, at a 10 µM concentration, regardless of MCP‐1 induction, Wj1113 exhibited a stronger inhibitory effect than tofacitinib, a commonly used JAK3 inhibitor serving as a positive control.

After MCP‐1 induction, we analyzed the mRNA expression of inflammatory cytokines in the migrated THP‐1 cells via real‐time quantitative polymerase chain reaction (RT‐qPCR). The heatmap (Figure 2F) demonstrated that Wj1113 significantly downregulated the mRNA （messenger ribonucleic acid） levels of key proinflammatory factors such as *Tnf‐α*, *Il‐6*, *Mcp‐1*, *Il‐1β*, *G‐csf*, and *Il‐23*, while simultaneously upregulating the expression of the anti‐inflammatory cytokine *Il‐10*. Notably, the secreted level of TNF‐α, which is highly relevant to arthritis, was significantly decreased after Wj1113 treatment (Figure 2E), and Wj1113's inhibitory effect surpassed that of the positive drug.

In conclusion, Wj1113 effectively suppresses monocyte chemotaxis and modulates inflammatory cytokine expression in monocytes, indicating its potential as a valuable therapeutic candidate for arthritis by targeting monocyte‐related mechanisms.

### 2.3 Wj1113‐Mediated Inhibition of Macrophage and B Cell Activation

**FIGURE 3:**
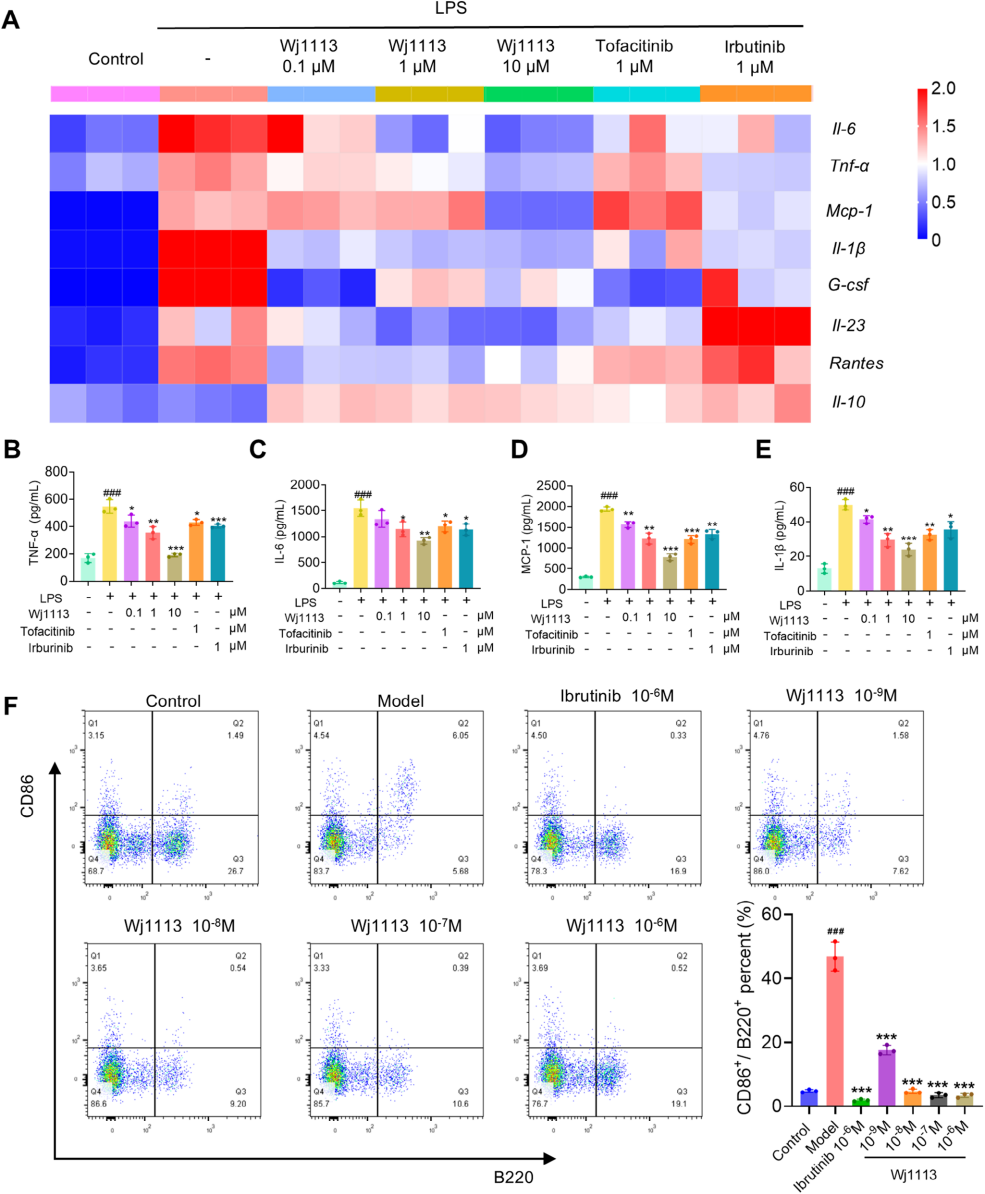
Effects of Wj1113 on macrophage and B cell activation. (A–E) Regulation of Wj1113 on inflammatory factors following macrophage activation. Primary peritoneal macrophages from mice were treated with LPS (100 ng/mL) and different concentrations of Wj1113 for 24 h. The mRNA expression of various inflammatory cytokines was analyzed by RT‐qPCR, with the results presented as a heatmap. Additionally, cytokine levels in cell culture supernatants were measured by ELISA. (F) Inhibitory effect of Wj1113 on B‐cell activation. Flow cytometry analysis was performed on activated B cells (CD86+/CD220+) in mouse splenic lymphocytes. The lymphocytes were treated with varying concentrations of Wj1113 for 48 h after IgM stimulation. The B‐cell activation ratio was calculated as (Q2/(Q2 + Q3)). Data are presented as means ± SD. Statistical significance is indicated as follows: ###*p* < 0.001 versus the control group; *p < 0.05, **p < 0.01, and ***p < 0.001 versus the LPS group or model group.

In arthritis pathogenesis, macrophage and B cell activation are crucial. For macrophage activation studies, we utilized primary mouse peritoneal macrophages and RAW264.7 murine macrophages. In primary macrophages, the RT‐qPCR heatmap (Figure 3A) revealed that LPS (lipopolysaccharide) enhanced the mRNA of proinflammatory cytokines like *Tnf‐α*, *Il‐6*, *Mcp‐1*, *Il‐1β*, *G‐csf*, and *Il‐23*, and *Rantes*, but decreased *Il‐10* mRNA. With Wj1113 treatment, the proinflammatory mRNA levels dropped significantly, and *Il‐10* mRNA increased. At 1 µM, Wj1113 surpassed tofacitinib and irbutinib in inhibiting *Il‐6*, *Il‐23*, and *Rantes*. Enzyme linked immunosorbent assay (ELISA) results (Figure 3B–E) also showed Wj1113 effectively curbed TNF‐α, IL‐6, MCP‐1, and IL‐1β secretion from activated macrophages. Similar outcomes were seen in RAW264.7 macrophages where Wj1113 suppressed proinflammatory mRNA and protein and boosted anti‐inflammatory ones (Figure S1).

Regarding B cell activation, flow cytometry post anti‐IgM(immunoglobulin M) induction showed that anti‐IgM activated B cells. However, 1 nM Wj1113 reduced the activation rate from 47 to 18%, and 10 nM Wj1113 completely inhibited it (Figure 3F).

In conclusion, Wj1113 strongly inhibits both macrophage and B cell activation, likely due to its modulation of relevant arthritis signaling pathways.

### 2.4 In Vivo Efficacy in CIA Mouse Arthritis Model of Wj1113

**FIGURE 4:**
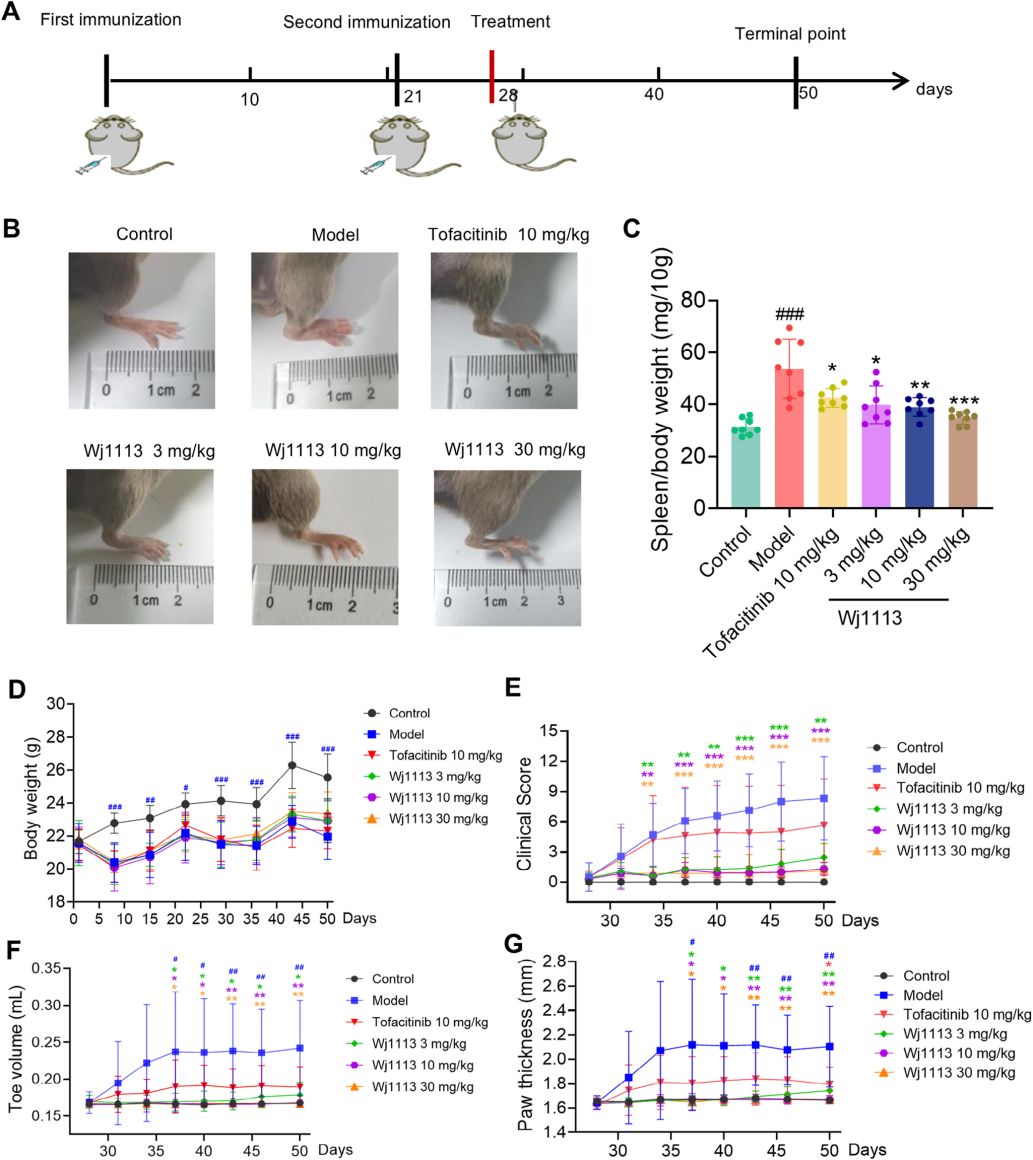
Wj1113 evaluation in CIA mouse model. (A) CIA modeling procedure. (B) Images of the mice's right hind paws post 3‐week Wj1113 treatment. (C) Spleen index of mice. (D) Mouse weight change curve. Clinical scores (E), toe volume (F), and paw thickness (G) of CIA mice in different groups starting from group dosing, measured every 3 days. Data from eight mice per group, and data are presented as means ± SD. #*p* < 0.05, ##*p* < 0.01, and ###*p* < 0.001 versus control group; *p < 0.05, **p < 0.01, and ***p < 0.001 versus CIA group.

Building on the remarkable in vitro performance of Wj1113, we embarked on an in‐depth exploration of its therapeutic potential within the context of the CIA mouse model. As per the experimental protocol depicted in Figure 4A, we conducted a series of efficacy experiments. Visual improvements were evident in the mice treated with Wj1113. Figure 4B, presenting the postmodeling paw photos of the mice, clearly shows that those administered with Wj1113 at doses of 3, 10, and 30 mg/kg exhibited a notable reduction in hind paw swelling and redness.

Upon measurement and scoring, we found significant dose‐dependent decreases in the clinical swelling scores of limb joints when comparing Wj1113‐treated groups with the model group. Specifically, at 10 mg/kg, the average clinical swelling score was 1.3, and at 30 mg/kg, it was 1.1, in contrast to the model group's score of 8.3 (Figure 4E).

Regarding toe volume, starting from the 10 mg/kg dose of Wj1113, the average volume was 0.168 mL, nearly equivalent to that of normal mice. In comparison, the model group had an average toe volume of 0.242 mL (Figure 4F). When it came to paw thickness, at a 10 mg/kg dose, the average paw thickness was 1.7 mm, which was the same as that of normal mice, while the model group had a thickness of 2.1 mm (Figure 4G).

Notably, at the 10 mg/kg dose, Wj1113 was capable of almost completely alleviating various joint‐related indicators in mice. Its performance at this dose was significantly more effective than that of the positive control drug, tofacitinib. Throughout the experiment, Wj1113 did not induce any changes in the body weight of the animals (Figure 4D). Furthermore, to assess the potential toxicity of Wj1113, we administered a high dose of the compound, specifically 300 mg/kg body weight, to the mice. As depicted in Figure S4, even when given a 10‐fold higher dose, Wj1113 did not affect the body weight of the mice (Figure S4A). Additionally, histological examination using hematoxylin and eosin (HE) staining of the liver, kidneys, and other organs revealed no pathological changes (Figure S4B,C). This finding, consistent with our previous toxicity studies, attests to its favorable safety profile [18].

After model induction, the spleen index of the mice increased significantly due to collagen‐induced immune stimulation. However, treatment with both tofacitinib and Wj1113 led to a significant dose‐dependent reduction in the spleen index. For example, at 10 mg/kg of Wj1113, the spleen index decreased by approximately 27% compared with the untreated model group, and at 30 mg/kg, it decreased by around 35% (Figure 4C). This outcome strongly suggests that Wj1113 has the potential to treat arthritis by modulating immune inflammation.

### 2.5 Wj1113 Significantly Ameliorates Joint Pathological Conditions in CIA Mice

To precisely assess the ameliorative effects of compound Wj1113 on mouse RA, histological analyses using HE staining and Fast Green‐Safranin O staining were conducted on the right hind paws of the mice.

**FIGURE 5:**
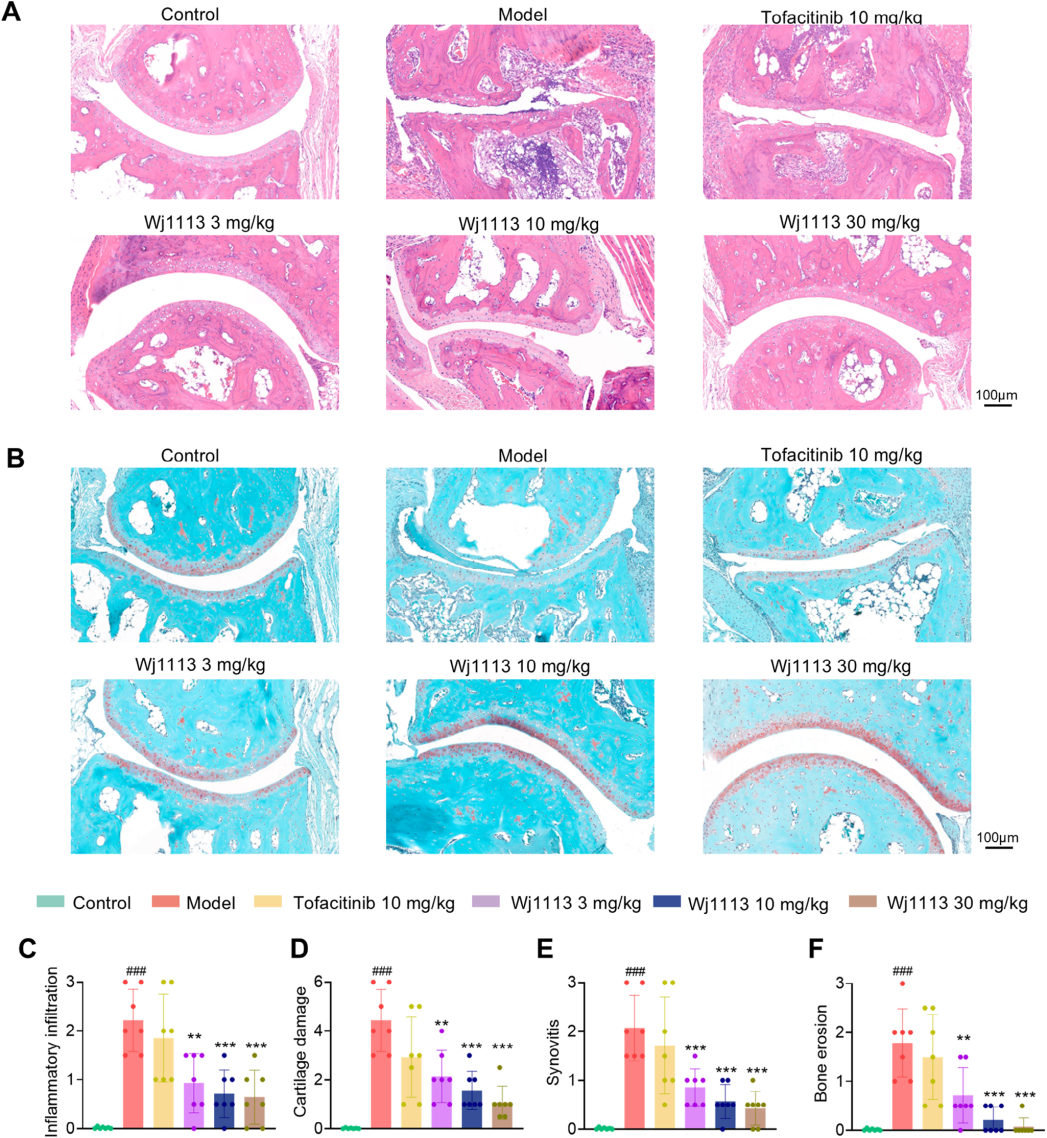
Histological analyses and quantitative evaluations. (A) H&E‐stained tissue sections for inflammation‐related scoring. Paraffin‐embedded joint sections were H&E‐stained. These images were used to score inflammatory cell infiltration (C). (B) Fast Red O/Alcian Blue‐stained sections for cartilage & bone scoring. Sections were stained with fast red O and counterstained with Alcian blue, enabling assessment of cartilage damage (D), and synovial inflammation (E) and bone erosion (F) by highlighting cartilage matrix and bone structures. Data from seven samples per group are presented as means ± SD. Statistical significance: ###*p* < 0.001 versus control; **p < 0.01, ***p < 0.001 versus model group.

HE staining results (Figure 5A,C) showed that in the CIA model group, the joint cavity had significant inflammatory cell infiltration, along with synovial hyperplasia and angiogenesis. Wj1113 treatment significantly reduced this infiltration and alleviated joint damage. Fast Green‐Safranin O staining (Figure 5B) revealed severe damage to the cartilage tissue at the joint cavity margins after model induction. Statistical analysis (Figure 5D–F) confirmed increased cartilage injury, bone erosion, and destruction. Wj1113 treatment led to dose‐dependent decreases in these indicators (Figure 5A–F), outperforming the positive control tofacitinib.

For a comprehensive view of joint protection, 3D micro‐CT scans were done. As seen in Figure S3, model group mice had enlarged and damaged joints. Tofacitinib (10 mg/kg) had limited effect. In contrast, Wj1113 at 3 mg/kg showed notable improvement, and at 10 mg/kg, joint damage was minimal.

These findings from staining and CT scans demonstrate Wj1113's significant therapeutic impact on arthritis.

### 2.6 Wj1113 Potently Modulates Inflammatory Factor Expression in CIA Mice

**FIGURE 6:**
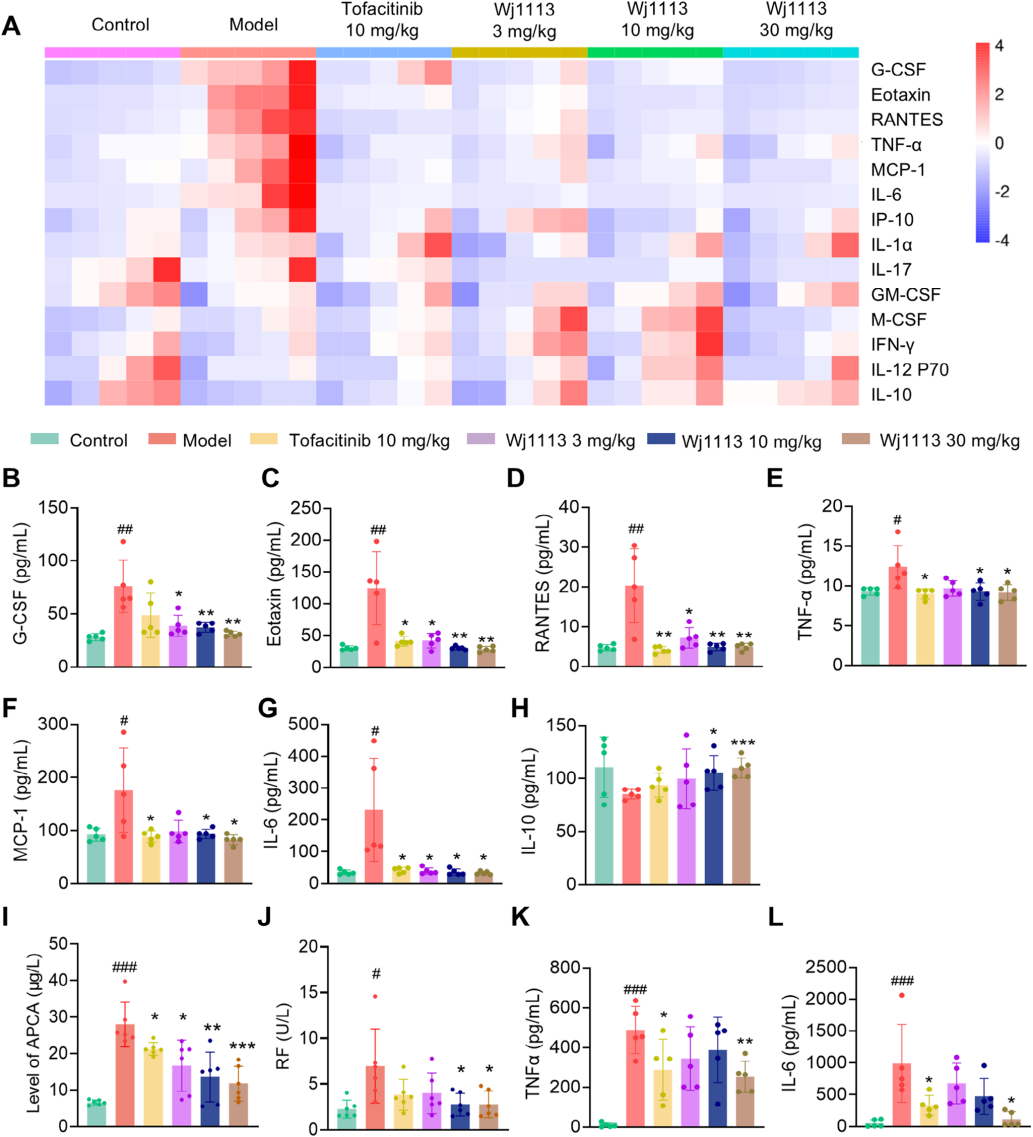
Inflammatory factors in CIA mouse joint tissue and serum. (A) Heatmap of multifactor detection in mouse joint tissue. (B–H) Statistical analysis of representative inflammatory factors from multifactor assay. (I and J) ELISA‐detected arthritis biomarkers in mouse joint tissue. (K and L) ELISA‐measured inflammatory factors in mouse serum. Data from five mice per group for multifactor detection and six mice per group for ELISA assay are presented as means ± SD. Statistical significance: #*p* < 0.05, ##*p* < 0.01, ###*p* < 0.001 versus control; *p < 0.05, **p < 0.01, ***p < 0.001 versus model group.

To delve deeper into the mechanism by which Wj1113 alleviates CIA, we employed a multiplex assay kit to examine the expression of inflammatory factors in the joint tissue posttreatment. As depicted in Figures 6A–H, the CIA model led to a substantial upregulation of chemokines such as G‐CSF, Eotaxin, MCP‐1, and RANTES, along with proinflammatory cytokines like IL‐6 and TNF‐α. In contrast, the anti‐inflammatory factor IL‐10 experienced a significant decline after model induction. Treatment with Wj1113, however, led to a dose‐dependent decrease in the overexpressed chemokines and proinflammatory factors. Simultaneously, the expression of IL‐10 was significantly enhanced. These results imply that Wj1113 may mitigate arthritis symptoms by suppressing chemokines and proinflammatory factors while boosting the expression of anti‐inflammatory factors, potentially through its modulation of the JAK3 and BTK signaling pathways.

We also measured the serum levels of two crucial inflammation‐related cytokines, TNF‐α and IL‐6, which are intricately linked to arthritis pathogenesis. In line with the findings in joint tissue, treatment with Wj1113 significantly decreased the serum levels of both TNF‐α and IL‐6 in a dose‐dependent manner (Figure 6K,L). Notably, at the same dose, Wj1113 outperformed the positive control drug, underscoring its potent anti‐inflammatory capabilities.

Furthermore, we assessed the influence of Wj1113 on specific arthritis biomarkers. As shown in Figure 6I,J, Wj1113 significantly reduced the levels of ACPA and RF, key indicators of arthritis severity. This additional evidence further validates the therapeutic potential of Wj1113 in managing and alleviating arthritis symptoms.

### 2.7 Wj1113 Dampens BTK and JAK3‐Related Signaling Pathways and Diminishes Macrophage Accumulation in Vivo

**FIGURE 7:**
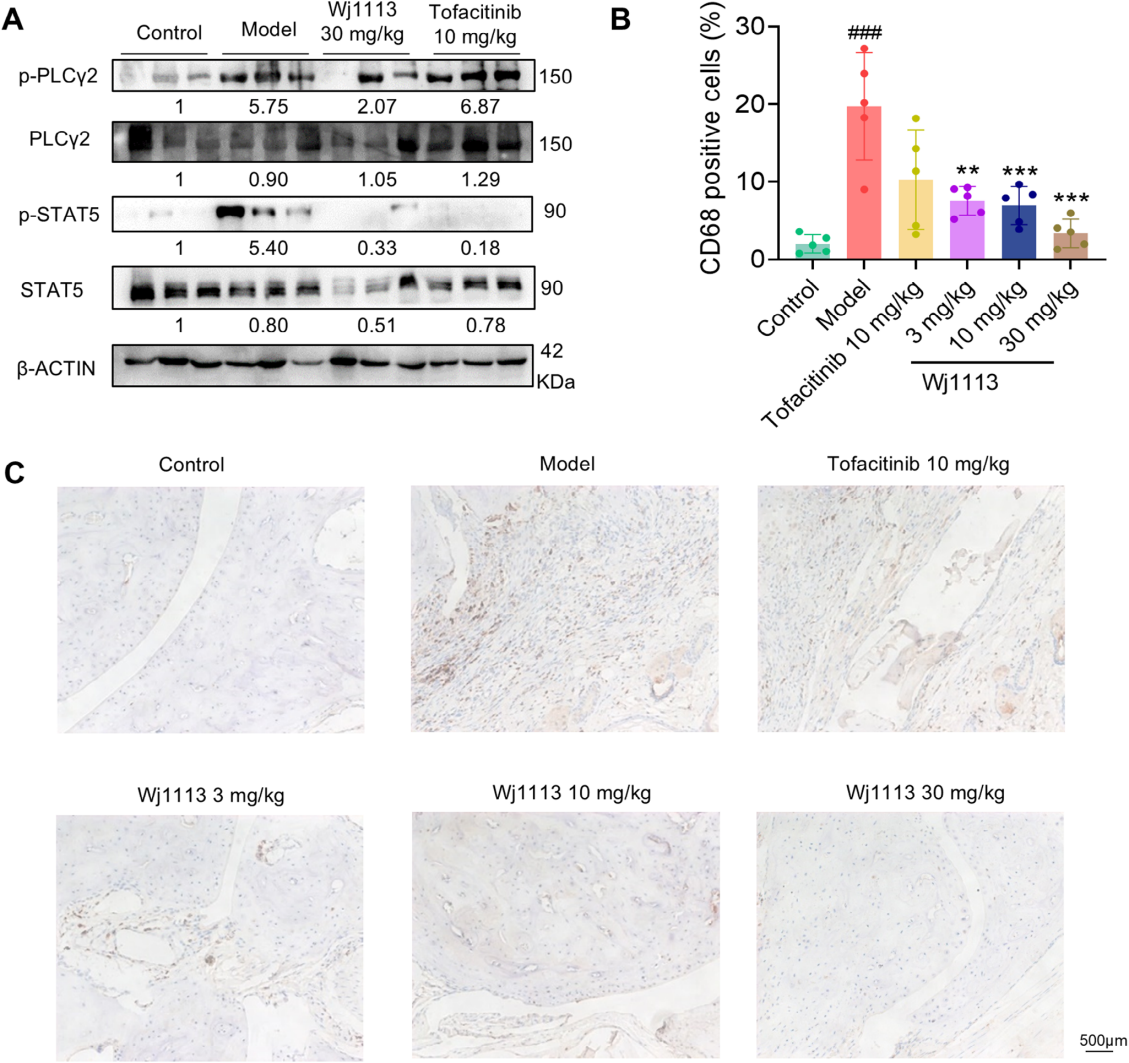
Effects of Wj1113 on JAK3/BTK signaling pathways and macrophage activation in vivo. (A) The influence of Wj1113 on the downstream signaling pathways of JAK3 and BTK. Western blot analysis of joint tissue, with data from three samples. (B) Quantitative analysis of macrophage (CD68+) counts in joint tissue sections. The data derived from immunohistochemical images shown in (C), are based on five samples. Data are presented as means ± SD. ###*p* < 0.001 versus the control group; **p < 0.01, and ***p < 0.001 versus the relevant group.

We explored the impact of compound Wj1113 on its target pathways within the joint tissues of mice. As depicted in Figure 7A, in the arthritis model tissues, the BTK and JAK3 signaling pathways were activated. This was evidenced by a substantial increase in the phosphorylation levels of the downstream proteins PLCγ2 and STAT5. However, upon treatment with Wj1113, the phosphorylation levels of these two proteins were significantly suppressed. This finding indirectly indicates that Wj1113 can effectively inhibit the activity of BTK and JAK3 proteins in the in vivo context.

To further elucidate the effect of Wj1113 on immune cells in the joint tissues, we conducted immunohistochemical staining to evaluate its influence on macrophages (Figure 7B,C). The results revealed a marked decrease in the number of macrophages accumulated in the arthritic tissue after treatment with Wj1113. This clearly shows that Wj1113 exerts its therapeutic effect on murine arthritis by reducing the accumulation of macrophages in the joint tissues, likely contributing to the alleviation of local inflammation.

## 3 Discussion

Over the past decade, remarkable progress has been made in RA treatment, with a shift from traditional approaches toward more targeted and precise therapeutic strategies. JAK3 has emerged as a promising therapeutic target, with its inhibitor, tofacitinib, already approved and available on the market. Meanwhile, BTK inhibitors are currently under clinical investigation, demonstrating potential in the treatment of RA. This highlights the significance of targeting these kinases in RA therapy. Recently, dual BTK/JAK3 inhibitors have drawn extensive attention in the fields of cancer and arthritis treatment. By simultaneously acting on these two crucial targets, these inhibitors hold the promise of modulating multiple pathways involved in the immune dysregulation of RA, potentially overcoming the limitations of single‐target therapies and offering better treatment outcomes.

Over the past decade, remarkable progress has been made in RA treatment, with a shift from traditional approaches toward more targeted and precise therapeutic strategies [21, 22]. JAK3 has emerged as a promising therapeutic target, with its inhibitor, tofacitinib, already approved and available on the market [20]. Meanwhile, BTK inhibitors are currently under clinical investigation, demonstrating potential in the treatment of RA [23]. Recently, dual BTK/JAK3 inhibitors have drawn extensive attention in the fields of cancer and arthritis treatment [24, 25]. By simultaneously acting on these two crucial targets, these inhibitors hold the promise of modulating multiple pathways involved in the immune dysregulation of RA, potentially overcoming the limitations of single‐target therapies and offering better treatment outcomes [24, 26]. This comprehensive approach may result in improved treatment outcomes and address the limitations associated with single‐target therapies.

In this study, we identified Wj1113 as a novel dual inhibitor of BTK and JAK3, presenting a promising therapeutic approach for RA. Our findings strongly demonstrated that Wj1113 effectively inhibits the BTK and JAK3 signaling pathways both in vitro and in vivo. The remarkable reduction in the phosphorylation levels of downstream targets PLCγ2 and STAT5 serves as clear evidence that Wj1113 can effectively inhibit the BTK and JAK3 signaling pathways. This inhibition is fundamental as it enables Wj1113 to precisely regulate the complex network of biological processes, which are central to the pathogenesis of RA.

Beyond its molecular effects, Wj1113 significantly reduced the chemotaxis of monocytes as well as the activation of macrophages and B cells. Monocytes and macrophages are critical mediators of joint inflammation and tissue destruction in RA. By inhibiting their recruitment and activation, Wj1113 may help mitigate cartilage and bone damage, addressing one of the key challenges in RA therapy, which is controlling inflammation and preventing long‐term joint degradation.

In the CIA mouse model, Wj1113 outperformed the JAK inhibitor tofacitinib, even at lower doses. This advantage is likely attributed to the dual inhibition of BTK and JAK3 by Wj1113, enabling it to comprehensively disrupt the complex immune networks driving RA progression. By targeting both B cell‐mediated and cytokine‐driven pathways, Wj1113 offers a broader and more potent therapeutic benefit, further emphasizing its potential as a treatment for RA.

In addition to investigating the mechanism and efficacy, we also evaluated the pharmacokinetic properties and safety profile of Wj1113, which strongly support its potential as a drug candidate. Our previous studies indicated that Wj1113 possesses excellent pharmacokinetic characteristics. With an oral bioavailability as high as 40.98%, it surpasses many reported covalent inhibitors. After intravenous administration, its half‐life (*t*1/2) is 0.59 h, comparable to other covalent compounds, which is beneficial for the drug's effective action in vivo [27, 28].

In terms of safety, Wj1113 also demonstrated promising results. The single‐dose acute toxicity study showed that the maximum tolerated dose in mice exceeded 500 mg/kg, indicating a wide safety margin. The human ether‐a‐go‐go related gene (hERG) assay revealed low cardiotoxicity, and the AMES test confirmed its nonmutagenicity, further validating its overall genetic safety. Moreover, our cytotoxicity assessment of the cell lines used in the experiments showed that even at a relatively high dose of 10 µM, Wj1113 had minimal inhibitory effects on the growth of THP‐1, RAW264.7, and mouse splenic lymphocytes. During the 50‐day treatment of mice with 30 mg/kg Wj1113, no adverse effects on body weight were observed, further confirming the compound's safety.

As a dual‐target inhibitor, Wj1113 offers distinct advantages over single‐target inhibitors and current first‐line arthritis treatments. First, it can act on monocytes, macrophages, and B cells simultaneously, providing a more comprehensive therapeutic effect than the positive control drug, tofacitinib. Second, due to its broader target spectrum, Wj1113 is less likely to induce drug resistance compared with single‐target kinase inhibitors. However, it is important to note that inhibiting two distinct targets simultaneously may lead to cumulative toxicity, potentially resulting in higher overall toxicity than single‐target drugs. This is an aspect that requires close attention in future research and clinical applications.

Drug resistance is a common challenge associated with the clinical use of kinase inhibitors, usually emerging over months to years. Existing literature indicates that resistance to JAK3 and BTK inhibitors may vary depending on specific mutations and underlying mechanisms [29, 30, 31]. For example, patients treated with JAK inhibitors may develop resistance due to mutations in the JAK2 kinase domain or the activation of alternative signaling pathways. Similarly, BTK inhibitors may face resistance due to mutations in the BTK gene or the activation of bypass signaling pathways. Based on the dual‐target mechanism of Wj1113, we speculate that its efficacy can be maintained for at least several months to years, as the dual‐target inhibition may slow down the development of drug resistance. However, specific experimental verification of this speculation is needed and cannot be achieved in the short term. If Wj1113 progresses to clinical trials, we will closely monitor the emergence of drug resistance as part of a comprehensive clinical evaluation strategy to provide relevant data for future use.

In conclusion, this study demonstrates that Wj1113, a novel dual BTK/JAK3 inhibitor, can effectively inhibit BTK and JAK3 kinases, suppress cytokine signaling, inhibit monocyte chemotaxis and macrophage activation, and block B cell activation. In the CIA mouse model, treatment with Wj1113 significantly improves arthritis symptoms, joint pathology, and the expression of inflammatory factors. These findings highlight the great potential of Wj1113 as a candidate for arthritis treatment. Further research is required to explore its clinical applications, potential combination therapies, and to address the identified limitations to fully realize its potential in treating RA and providing better treatment options for patients.

## 4 Method

### 4.1 Cell Culture

The human acute monocytic leukemia cell line THP‐1 and the mouse macrophage cell line RAW264.7 were sourced from the Cell Center of the Institute of Basic Medical Sciences, Chinese Academy of Medical Sciences. THP‐1 cells were cultured in RPMI 1640 medium and RAW264.7 in high‐glucose DMEM, both supplemented with 10% FBS and 1% penicillin–streptomycin (100×). These cell lines were incubated in a humidified 37°C incubator with 5% CO₂. Once they reached ∼80% confluence, cells were passaged. Only those in the logarithmic growth phase were collected for subsequent experiments to ensure optimal physiological state and reliable results. All cell lines used in this study were identified by short tandem repeat analysis and mycoplasma cytoplasma test is negative.

### 4.2 Extraction of Mouse Splenic Lymphocytes and Isolation of Mouse Peritoneal Macrophages

SPF‐grade male C57BL/6J mice (6–8 weeks old), purchased from Huafukang Biological Technology Co., Ltd. (Beijing; license number SCXK (Jing) 2019‐0008), were used for both splenic lymphocyte extraction and peritoneal macrophage isolation. All procedures were carried out under aseptic conditions.

For splenic lymphocyte extraction, euthanized mice had their spleens dissected. The spleen tissues were ground in 5 mL of separation solution from the Mouse Lymphocyte Separation Kit (Dakewe Biotech) as per the kit's instructions. The homogenate was transferred to a tube, and 1 mL of serum‐free RPMI 1640 medium (Livning Company) was added on top. The tube was centrifuged at 800×*g* for 30 min at room temperature. The lymphocyte layer in the middle was collected and resuspended for cell counting.

For peritoneal macrophage isolation, after euthanasia, the abdominal skin of the mice was cut to expose the peritoneum. PBS was injected into the peritoneal cavity. The abdomen was gently massaged for 5 min to dislodge cells. The peritoneal lavage fluid was collected with a syringe and centrifuged at 450×*g* for 10 min at 4°C. The cell pellet was washed twice with PBS and resuspended in RPMI‐1640 medium. The cells were plated and incubated overnight. Subsequently, nonadherent cells were washed away with medium, leaving the adherent peritoneal macrophages.^32^

### 4.3 Western Blot Analysis

Collected cells were lysed with high‐efficiency RIPA lysis buffer (Solarbio, containing 1% protease and 1% phosphatase inhibitors). Lysates were sonicated at 40 W. Afterward, cell lysates were subjected to electrophoresis. Proteins were transferred to membranes, which were incubated with primary and then secondary antibodies. The blots were visualized. ImageJ was used to analyze the blots. Statistical values were annotated below the bands.

### 4.4 THP‐1 Cell Chemotaxis Assay

THP‐1 cells were treated with solvent control or different concentrations of Wj1113 (1, 10 µM) and incubated for 48 h. The bottom of a transwell plate was pretreated by coating with 30 µL of 20 µg/mL fibronectin (MedChemExpress).

For the MCP‐1 induced chemotaxis part: Collected cells were seeded into the transwell chamber at a density of 1 × 10⁵ cells/well. They were then incubated for 3.5 h in the bottom plate containing 600 µL of RPMI 1640 complete medium with 250 ng/mL MCP‐1 (MedChemExpress).

For the non‐MCP‐1 induced chemotaxis (cell supernatant effect) part: Untreated THP‐1 cells (1 × 10⁵ cells/well) were placed in the transwell chamber. The collected treated cell supernatant was added to each well of the bottom plate, and the cells were incubated for 24 h.

After incubation, migrated cells on the bottom side of the filter were fixed with 4% paraformaldehyde and stained with crystal violet. Microscopic images were taken, and the cells were counted.

### 4.5 Cell Proliferation Assay

Cell proliferation was assessed using the CellTiter‐Glo assay kit (Promega). Briefly, 30 µL reagent was added to each well. The plate was then shaken for 5 min in the dark. Luminescent signals were detected with an Envision instrument. GraphPad Prism 8.0 was used to calculate the IC_50_ values and plot the proliferation inhibition curve.

### 4.6 RT‐qPCR Experiment

The collected cells were washed with PBS. Subsequently, total RNA was extracted using an RNA fast extraction kit (Shanghai Yishan Biotechnology). Reverse transcription was carried out using the TransScript All‐in‐One First‐Strand cDNA Synthesis SuperMix for qPCR kit (Beijing TransGen Biotech). Finally, RT‐qPCR was performed with the Hieff qPCR SYBR Green Master Mix kit (Yeasen Biotech).

### 4.7 Flow Cytometry

Mouse splenic lymphocytes were treated with 30 µg/mL IgM (B cell activator, Southern Biotech) and incubated for 1 h. Then, the cells were treated with Wj1113 at different concentrations for 24 h. After centrifugation and cell collection, the cells were incubated with anti‐B220‐APC and anti‐CD86‐PE flow cytometry antibodies (BioLegend) for 25 min. Flow cytometry analysis was performed using FlowJo_10.8.1 software for data processing. The percentage reported was the proportion of activated B cells (Q2 cells) to total B cells (Q2+Q3 cells), calculated as Q2/ (Q2+Q3).

### 4.8 ELISA Assay

In a 96‐well ELISA plate, 100 µL sample was added to the well for 90 min incubation.

Afterward, the liquid was discarded, and 100 µL of biotinylated antibody working solution was added for another 60 min incubation. The wells were washed, followed by adding 100 µL of HRP‐conjugated working solution and incubating for 30 min. Finally, 90 µL of substrate solution was added to the washed well. The reaction was halted by adding 50 µL of stop solution. The absorbance at 450 nm was measured, and sample concentrations were calculated using the standard curve.

### 4.9 JAK3 kinase Inhibition Activity Assay

The DP‐Glo assay kits (Promega Corporation) were used as per the manufacturer's instructions. In a 384‐well assay plate, 1 mL compound solution, 2 µL JAK3 kinase, and 2 µL Substrate/ATP mixture were added per well. The plate was incubated at room temperature for 60 min. Then, 5 mL of ADP‐Glo Reagent was added to each well, and the plate was incubated for an additional 40 min. Next, 10 µL of Kinase Detection Reagent was added, followed by a 30‐min incubation. Luminescence signals were recorded using an EnVision plate reader (PerkinElmer, USA).

### 4.10 Immunohistochemistry

Paraffin‐embedded joint tissue sections were prepared and subsequently deparaffinized. After performing antigen retrieval, endogenous peroxidase was blocked. The sections were then blocked using 5% BSA for 30 min. Primary antibodies were applied, and the sections were incubated overnight at 4°C. On the following day, a secondary antibody was added for an additional 30 min. DAB substrate solution was utilized for staining development. The sections were counterstained with hematoxylin for 1 min. Following dehydration and air‐drying procedures, the sections were mounted using neutral gum. They were examined under a microscope, and images were captured for further analysis.

### 4.11 CIA Model Construction and Evaluation

Male DBA/1 mice (6–8 weeks old) also purchased from Huafukang, were bred in the Experimental Animal Center of the Institute of Materia Medica, Chinese Academy of Medical Sciences. 100 µg of Immunization Grade Chick Type II Collagen Solution (Chondrex) and Complete Freund's Adjuvant (Chondrex) were mixed 1:1 and ultrasonically emulsified for the modeling adjuvant. 0.1 mL of this or physiological saline was injected at the tail root of anesthetized mice. A second immunization with 50 µg of mixed emulsion near the initial site was done 21 days later.^33^

Drug administration began 4 weeks after the first injection, with mice grouped in eight per group and drugs given orally once daily. The model and blank groups received physiological saline. From model onset, mice's body weight was monitored weekly. Joint swelling was scored 0–4 every 3 days, higher scores indicating worse joint inflammation. The sum of limb scores was the clinical arthritis score. Hind paw thickness was measured with a digital caliper, and toe volume with a YLS‐7C meter, with averages calculated. After the experiment, mice were euthanized, spleens weighed, and the spleen index calculated (spleen index = spleen weight (mg)/mouse weight (10 g))

### 4.12 Mouse Joint CT

The left hind paws of the mice were fixed using 4% paraformaldehyde. The ankles of the mice were scanned by the Skyscan1276 Micro CT scanner software at 60 kV and 200 µA, with a scanning resolution of 6.5 µm. After reconstruction, the calcaneus was vertically aligned, and the tarsal bone region within 5.5 mm from the bottom of the calcaneus was defined as the region of interest. Three‐dimensional image reconstruction was carried out using N‐Recon software, followed by three‐dimensional analysis performed with CT‐AN software.

### 4.13 Mouse Joint Tissue Pathological Analysis

The joints of the mice were fixed in paraffin and then sectioned. HE staining as well as Fast Green‐Safranin O staining were conducted, and subsequently, microscopic photography was performed. After staining, the joint pathological images were analyzed by means of CaseViewer software to evaluate the severity of arthritis. The degree of inflammatory cell infiltration was evaluated as follows: 0 = no inflammatory cells; 1 = mild infiltration; 2 = moderate infiltration; 3 = severe infiltration. Cartilage damage was assessed in accordance with the Osteoarthritis Research Society International criteria.

### 4.14 Multiplex Analysis of Mouse Joint Tissues

The mice joints were collected and homogenized. The procedure using a multiplex assay kit (Millipore) was as follows: First, 25 µL samples was added to a pretreated 96‐well plate. 25 µL magnetic beads was then added, and the plate was incubated overnight at 4°C with gentle shaking. Next, the wells were washed twice using wash buffer. Detection antibodies were added, and the plate was incubated a in the dark for 1 h. After two washes, 25 µL of SA‐PE was added, and the plate was incubated in the dark for 30 min. Finally, detection was carried out using the Luminex MAGPIX with xPONENT software.

### 4.15 Statistical Analysis

All statistical analyses were performed using GraphPad Prism 8.0 software (GraphPad Software). Two‐way analysis of variance (ANOVA) followed by Tukey's post hoc test was used for clinical arthritis scores. One‐way ANOVA followed by appropriate post hoc tests was used for other experiments. A *p* value < 0.05 was considered statistically significant.

## Author Contributions

**Chunyu Zhang**, **Fangfang Lai,** and **Hang Gong** conducted most of the experiments and wrote the manuscript. **Shuying Li**, **Nan Xiang**, and **Liuyi Que** helped the animal experiment. **Nina Xue** assisted in the cell experiment. **Mengyao Hao** and **Enjia Zhou** contributed to the data analysis. **Xiaojian Wang** synthesized the compound Wj1113. **Taigang Liang** helped to revise the manuscript. **Jing Jin** designed and supervised the whole experiment. All authors have given approval to the final version of the manuscript.

## Ethics Statement

All experimental procedures were strictly conducted in accordance with the guidance policies of the Animal Ethics Committee of the Institute of Materia Medica, Chinese Academy of Medical Sciences. The reference number for the approval is 00005646.

## Conflicts of Interest

All authors declare no conflicts of interest.

## Supporting information



Supporting Information

## Data Availability

The raw data, the processed data and analysis scripts are available from the corresponding author upon reasonable request. And all experiments were conducted in accordance with relevant guidelines and regulations.
