# Supplementary material for: A Novel Dual Bruton's Tyrosine Kinase/Janus Kinase 3 Inhibitor Wj1113 and its Therapeutic Effects on Rheumatoid Arthritis
Source: MedComm (2020). 2025 Jul 7;6(7):e70207. doi: 10.1002/mco2.70207 (PMC12231052; doi:10.1002/mco2.70207)
Supplement: Supplementary file 1 — Supporting Information [file MCO2-6-e70207-s001.docx]

**A Novel Dual Bruton's tyrosine kinase (BTK)/Janus kinase 3(JAK3) Inhibitor Wj1113** **and Its Therapeutic Effects on Rheumatoid Arthritis**

Chunyu Zhang^1a, b^, Fangfang Lai^1a^, Hang Gong^1a^, Shuying Li^a^, Nan Xiang^c^, Liuyi Que^d^, Nina Xue^a^, Mengyao Hao^a^, Enjia Zhou^a^, Xiaojian Wang*^a^, Taigang Liang*^b^, Jing Jin*^a^

^a^State Key laboratory of Bioactive Substances and Functions of Natural Medicines, Institute of Materia Medica, Chinese Academy of Medical Sciences and Peking Union Medical College, Beijing, 100050, China

^b^ School of Pharmaceutical Science, Medicinal Basic Research Innovation Center of Chronic Kidney Disease, Ministry of Education, Shanxi Medical University, Taiyuan 030001, China

^c^ Key Laboratory of Molecular Pharmacology and Drug Evaluation (Yantai University), Ministry of Education; Collaborative Innovation Center of Advanced Drug Delivery System and Biotech Drugs in Universities of Shandong, Yantai University, Yantai 264005, China.

^d^Department of Oncology, The Second Hospital of Shanxi Medical University, Taiyuan,030001, China

^1^Co-first authors

*Co-corresponding authors: Xiaojian Wang, Taigang Liang and Jing Jin

1 Xiannongtan Street, Xicheng District, Beijing, China, 100050.

Tel.: +86 010 63165207; fax: +86 010 863165207

Email addresses: [rebeccagold@imm.ac.cn](mailto:rebeccagold@imm.ac.cn)

**Appendix A. Supplementary data**

**The following is Supplementary data to this article:**

**Figure S1**


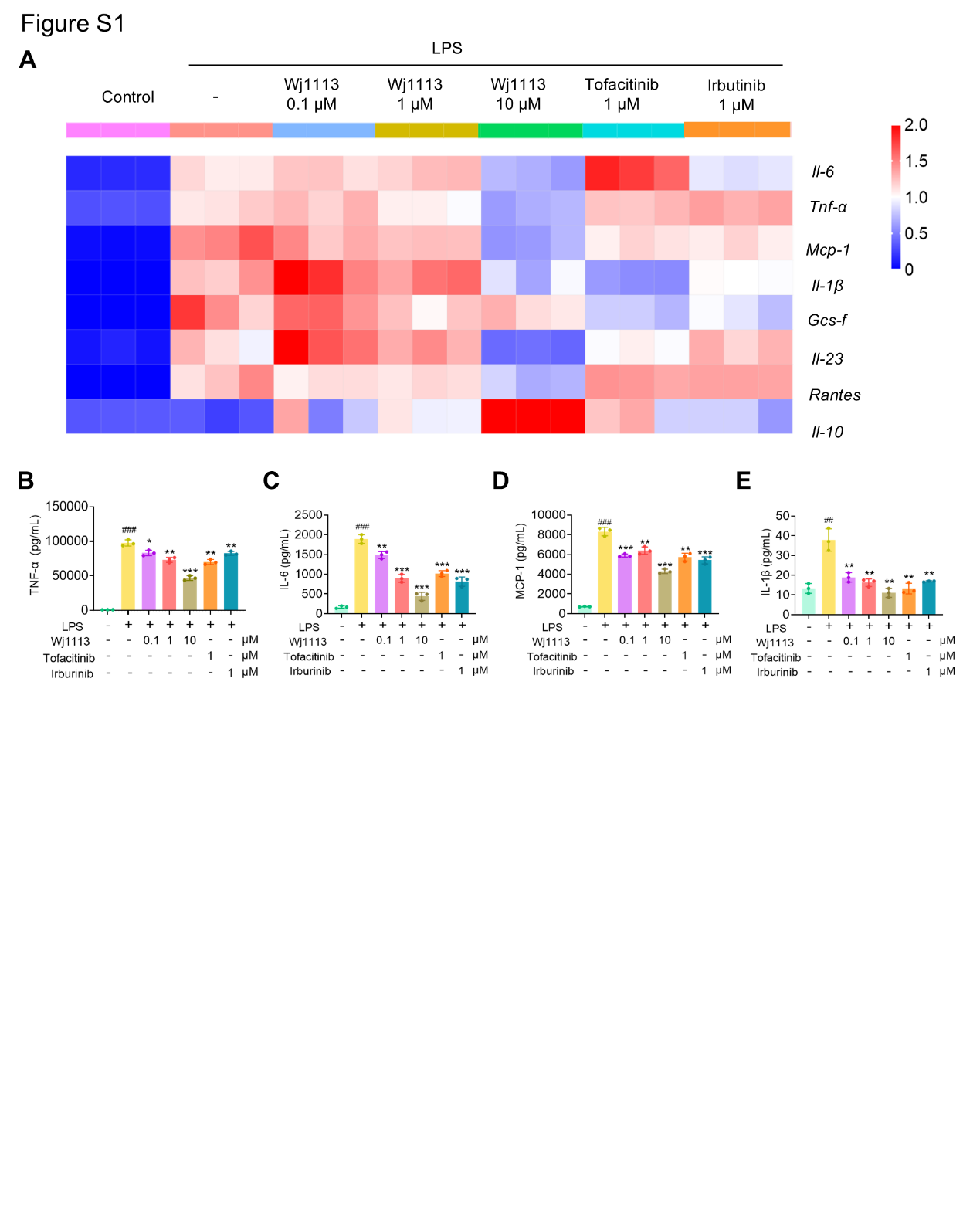


**Figure S2**


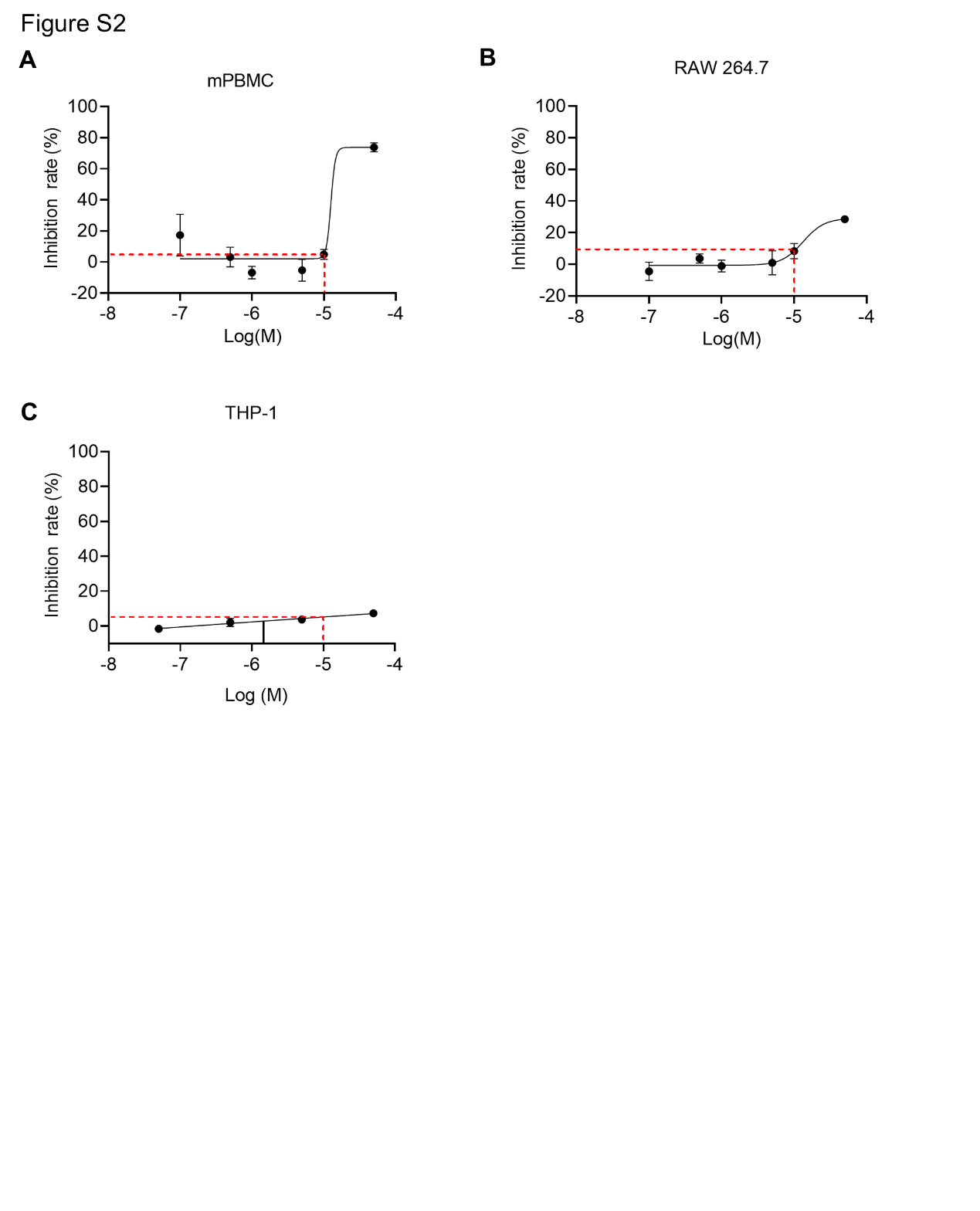


**Figure S3**


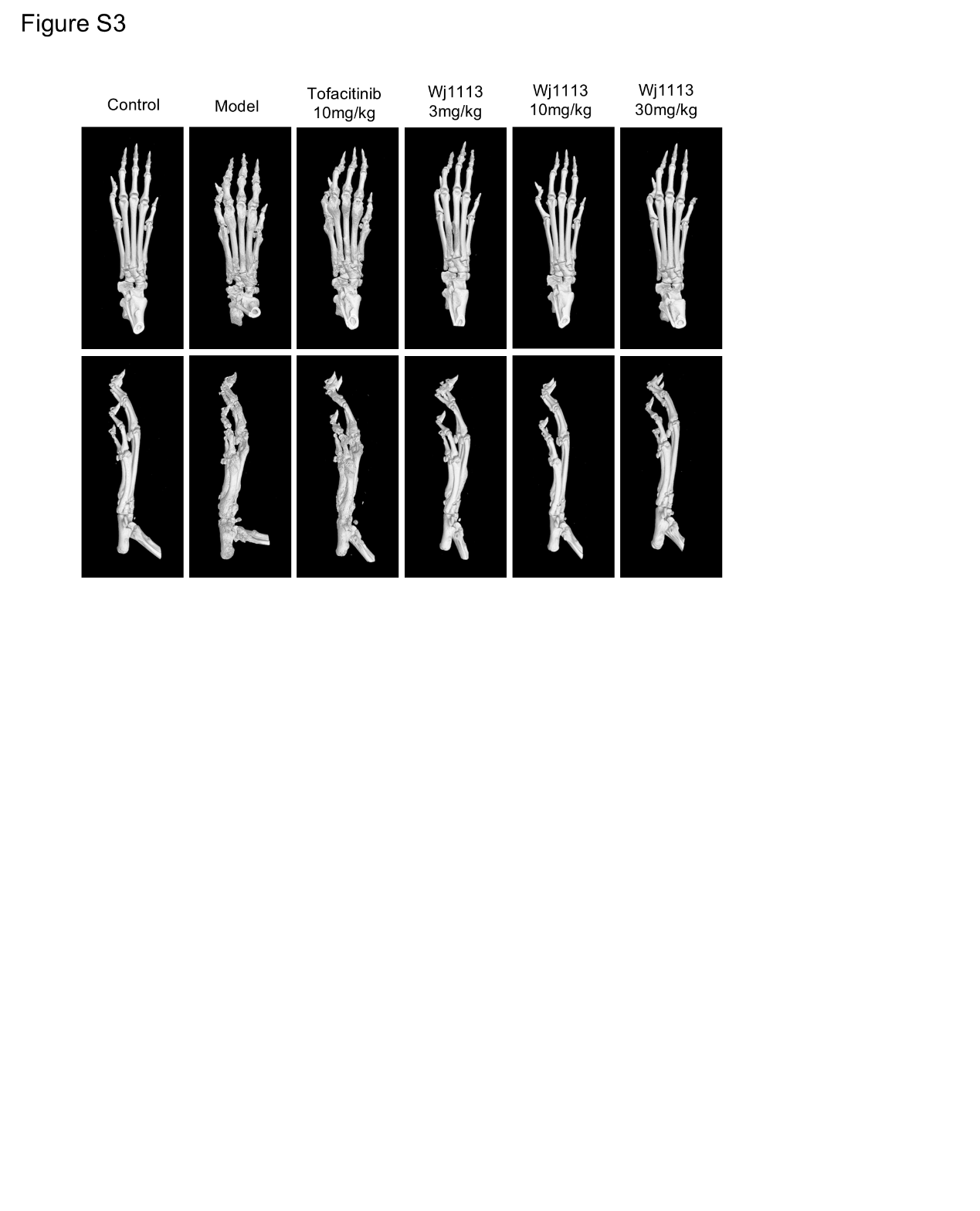


**Figure S4**


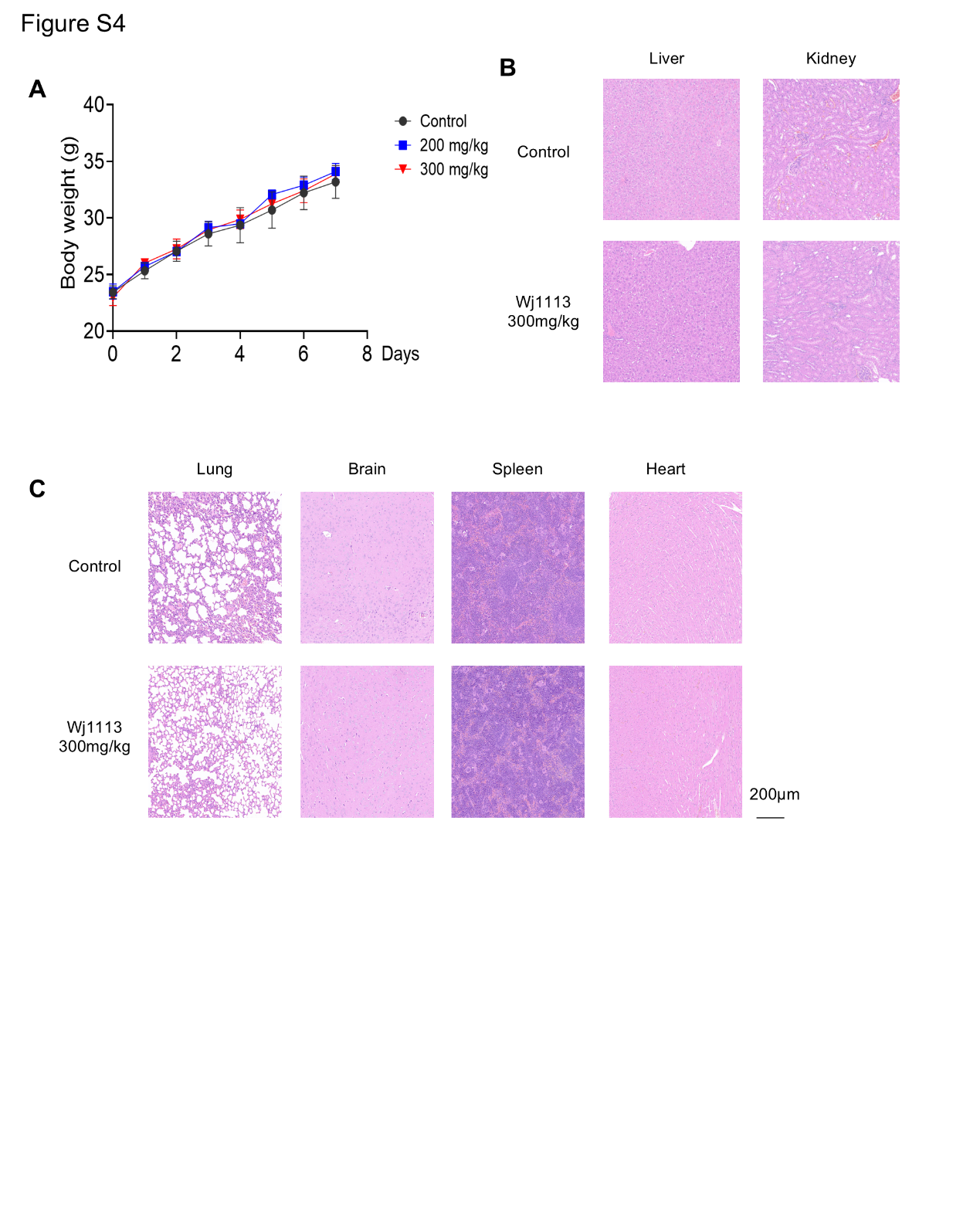


**Figure S1: Impact of Wj1113 on the activation of macrophage cell Line RAW264.7**

(A) Influence of Wj1113 on the mRNA expression of cytokines in activated RAW264.7 Cells. Macrophages were incubated with LPS (100 ng/mL) and varying concentrations of Wj1113 for 24 hours. The mRNA expression of multiple inflammatory cytokines was analyzed via RT-qPCR, and the outcomes were presented as a heatmap. (B-E) Cytokine levels in the cell culture supernatants were determined by ELISA. Data are presented as means ± SD. Statistical significance is indicated as follows: ##P < 0.01, ###P < 0.001 versus the control group; *P < 0.05, **P < 0.01, and ***P < 0.001 vs the LPS group.

**Figure S2: Cytotoxic effects of Wj1113 on different cell types.** (A) CTG assay measuring the cytotoxicity of Wj1113 on mouse lymphocytes after 48 hours of treatment. (B) Cytotoxic effects of Wj1113 on RAW264.7 cells after 24 hours of treatment. (C) Cytotoxic effects of Wj1113 on THP-1 cells after 24 hours of treatment. Data are presented as means ± SD.

**Figure S3:** **Representative micro-CT images of mouse CIA model in the right hind paw and knee**, n=1.

**Figure S4: In-vivo toxicity of a single high-dose administration of Wj1113**

(A) Body Weight Curve of Mice. Mice were administered a single dose of 200 or 300 mg/kg of Wj1113, and were sacrificed on the seventh day post-administration. (B) Toxicity of Wj1113 on Mouse Liver. Mouse livers were fixed with formalin, followed by H&E staining, and then photographed. (C) Toxicity of Wj1113 on Mouse Kidney. Mouse kidneys were fixed with formalin, underwent H&E staining, and were then photographed. N=5, Data are presented as means ± SD.
